# Cross-Disciplinary Approach of Adrenal Tumors: Insights into Primary Aldosteronism-Related Mineral Metabolism Status and Osteoporotic Fracture Risk

**DOI:** 10.3390/ijms242417338

**Published:** 2023-12-11

**Authors:** Alexandra-Ioana Trandafir, Ana-Maria Gheorghe, Oana-Claudia Sima, Adrian Ciuche, Eugenia Petrova, Claudiu Nistor, Mara Carsote

**Affiliations:** 1 PhD Doctoral School, Carol Davila University of Medicine and Pharmacy, 020021 Bucharest, Romania; alexandratrandafir26@gmail.com (A.-I.T.); oanaclaudia1@yahoo.com (O.-C.S.); 2 Department of Endocrinology, C.I. Parhon National Institute of Endocrinology, 011683 Bucharest, Romania; 3 Department 4—Cardio-Thoracic Pathology, Thoracic Surgery II Discipline, Faculty of Medicine, Carol Davila University of Medicine and Pharmacy, 020021 Bucharest, Romania; adrianciuche@gmail.com; 4 Thoracic Surgery Department, “Dr. Carol Davila” Central Emergency University Military Hospital, 010825 Bucharest, Romania; 5 Department of Endocrinology, Faculty of Medicine, Carol Davila University of Medicine and Pharmacy, 020021 Bucharest, Romania; jekined@yahoo.com (E.P.); carsote_m@hotmail.com (M.C.); 6 Clinical Endocrinology Department, C.I. Parhon National Institute of Endocrinology, 011683 Bucharest, Romania

**Keywords:** adrenal, primary aldosteronism, aldosterone, bone, osteoporosis, DXA, fracture, trabecular bone score, parathormone, bone turnover markers

## Abstract

Our objective was to overview the novel aspects in the field of adrenal gland neoplasms, namely, the management of bone status with respect to primary aldosteronism (PA). In the current narrative review, a PubMed study was conducted from inception until June 2023. The inclusion criteria were: human (clinically relevant) studies of any study design (at least 10 patients per study); English papers; and the following combination of key words within the title and/or abstract: “aldosterone” AND “bone”, “skeleton”, “osteoporosis”, “fracture”, “calcium”, “parathyroid”, “DXA”, “osteocalcin”, “P1NP”, “alkaline phosphatase”, “bone marker”, “trabecular bone score”, or “FRAX”. The exclusion criteria were in vitro or animal studies, reviews, and case reports/series. We screened 1027 articles and finally included 23 studies (13 of case-control type, 3 cross-sectional, 5 prospective, 1 observational cohort, and 1 retrospective study). The assessments provided in these studies were as follows: nine studies addressed Dual-Energy X-ray Absorptiometry (DXA), another study pointed out a bone microarchitecture evaluation underlying trabecular bone score (TBS), and seven studies investigated the bone turnover markers (BTMs) profile. Moreover, 14 studies followed the subjects after adrenalectomy versus medical treatment, and 21 studies addressed secondary hyperparathyroidism in PA patients. According to our study on published data during a period of almost 40 years (n = 23, N = 3965 subjects aged between 38 and 64, with a mean age 56.75, and a female-to-male ratio of 1.05), a higher PTH in PA versus controls (healthy persons or subjects with essential hypertension) is expected, secondary hyperparathyroidism being associated in almost half of the adults diagnosed with PA. Additionally, mineral metabolism anomalies in PA may include lower serum calcium and higher urinary calcium output, all these three parameters being reversible under specific therapy for PA, regardless medical or surgical. The PA subgroup with high PTH seems at higher cardiovascular risk, while unilateral rather than bilateral disease was prone to this PTH anomaly. Moreover, bone mineral density (BMD) according to central DXA might show a higher fracture risk only in certain adults, TBS being a promising alternative (with a still unknown perspective of diabetes’ influence on DXA-TBS results in PA). However, an overall increased fracture prevalence in PA is described in most studies, especially with respect to the vertebral site, the fracture risk that seems correctable upon aldosterone excess remission. These data recommend PA as a cause of secondary osteoporosis, a treatable one via PA intervention. There is still an area of debate the way to address BMTs profile in PA, the case’s selection toward specific bone evaluation in every day practice, and further on, the understanding of the potential genetic influence at the level of bone and mineral complications in PA patients.

## 1. Introduction

Primary aldosteronism (PA), also known as Conn syndrome, characterized by renin-independent aldosterone secretion, represents the most common cause of secondary hypertension among middle-aged adults. PA is either sporadic or familial, being classified into two major subtypes: unilateral (traditionally named an aldosterone-producing adenoma, responsible for 50–60% of cases) and bilateral (bilateral adrenal hyperplasia/disease involving 30–35% of all patients diagnosed with PA), as well as rare forms (unilateral hyperplasia, adrenocortical carcinoma, or familial hyperaldosteronism) [1,2]. The 2022 WHO (World Health Organization) classification recommended an immunohistochemistry analysis (HISTALDO) in addition to the traditional hormonal assessment in order to describe CYP11B2 (aldosterone synthase) activity amid adrenal lesions with potential aldosterone excess involvement [3].

The prevalence of PA depends on the studied population ranging from 3.2–12.7% in primary care centers to 29.8% in specialized referral hospitals [4,5], respectively, 5–10% among hypertensive individuals (almost 20% of individuals with resistant hypertension to standard blood-pressure-lowering medication have PA) [6,7,8].

Various pathogenic variants have been detected in PA, including somatic mutations, such as *KCNJ5*, *CACNA1D, ATP1A1*, *ATP2B3*, and *CTNNB1* genes [9,10]. The algorithm of approaching PA is based on successive tests (screening followed by confirmatory tests), as well as imaging tests for uni/bilateral disease (including adrenal vein sampling in selected cases) [11]. Screening protocols (aldosterone-to-renin ratio) are recommended for a selective subgroup, e.g., in cases with standard therapy-resistant hypertension, high blood and hypokalemia, hypertension onset at <40 years, people with a family history of hypertension or stroke at a young age, atrial fibrillation, and incidental adenoma. After a positive screening test, confirmatory tests such as the fludrocortisone suppression test, oral sodium loading test, saline infusion test, and captopril challenge test are required depending on the patient [12,13,14].

Persistent aldosterone excess plays a key role in cardio-renal injury and the development of cardio-metabolic complications, through the pro-fibrotic, pro-inflammatory effects, oxidative stress, and insulin resistance [15,16,17]. Untreated PA is associated with an increased morbidity due to coronary artery disease, left ventricular hypertrophy, atrial fibrillation, heart failure, stroke, diabetes mellitus, and obstructive sleep apnea that contribute to an overall decreased quality of life and a higher mortality rate than the general population [18,19,20,21].

PA management includes adrenalectomy (nowadays, laparoscopic access is preferred to open surgery) in unilateral forms and medical treatment with mineralocorticoid receptor antagonists (MRAs) such as spironolactone and eplerenone in bilateral disease in addition to the multidisciplinary management of comorbidities. New therapeutic perspectives are under development such as macrophage antibiotics or aldosterone synthase inhibitors [22,23,24,25].

**Figure 1:**
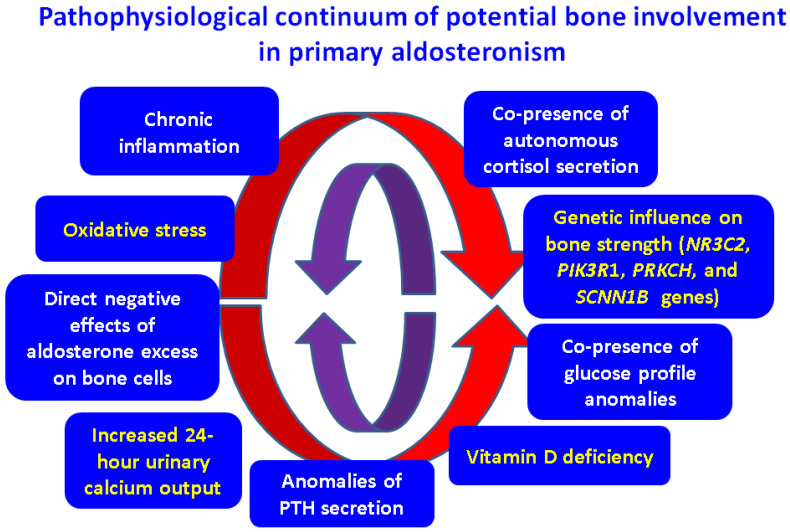
Potential common pathogenic traits in bone anomalies with respect to PA patients [26,27,28,29,30,31,32,33,34,35,36,37].

Recently, it has been recognized that mineralocorticoids may also play a role in bone turnover. Some authors suggest that an excess of aldosterone may be a cause of secondary osteoporosis [26,27]. The underlying mechanisms involve oxidative stress, inflammation, and a potential genetic influence concerning bone strength through various genes like *NR3C2*, *PIK3R1*, *PRKCH*, and *SCNN1B* [28,29]. Another pathway relates to aldosterone-mediated increased 24 h (hours) urinary calcium and consecutive secondary hyperparathyroidism in addition to a low vitamin D status [30,31]. Animal models showed that MRA might induce a bone strength recover in PA [32,33]. In humans, MRs have been identified at the level of bone cells, confirming that aldosterone has a direct effect on bone metabolism [34,35]. Moreover, MRs expression has been confirmed in parathyroid glands, suggesting that aldosterone may directly regulate parathyroid hormone (PTH) synthesis and secretion [36,37] (Figure 1).

However, which is the exact extend of bone involvement in PA still represents an open issue. Moreover, the practical importance of assessing the bone status in PA in order to address a specific intervention against osteoporosis and fracture risk reduction is less clear representing a topic of individual decision rather than large screening protocols and it should be taken into consideration other contributors, as well, such as age, menopausal status, concurrent diseases that are considered prone to fragility fractures, etc.

**Aim**. Our objective was to overview published data concerning the bone status, including mineral metabolism and fracture risk evaluation, in patients confirmed with adrenal tumors underling PA. The synopsis of data followed several aspects such as DXA results in PA patients, TBS in these subjects, the bone turnover markers profile, prevalent fragility fractures, as well as the assessment of mineral metabolism, particularly PTH assays.

**Figure 2:**
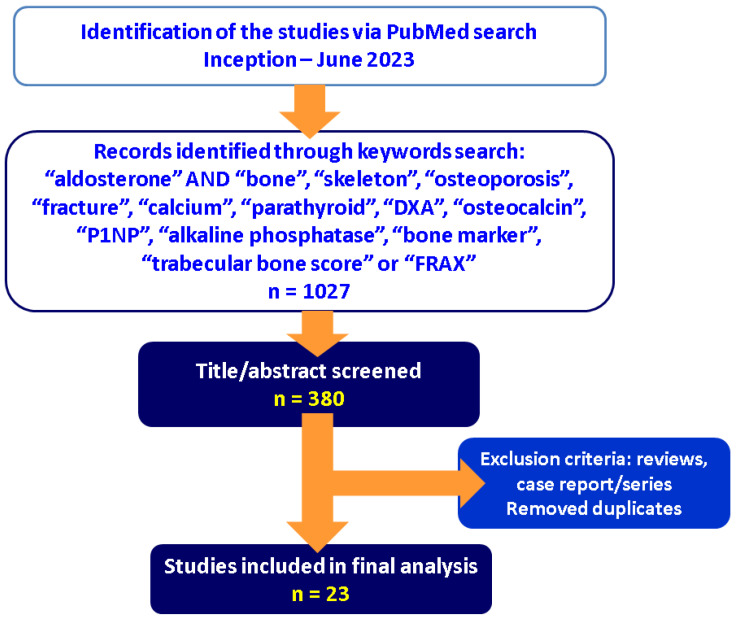
Flowchart of research (abbreviations: n = number of articles).

**Methodology.** This is a narrative review. A PubMed study was conducted with regard to the bone profile in PA from inception until June 2023. Inclusion criteria were: human (clinically relevant) studies of any study design (at least 10 patients per study); English papers; the following combination of key words within the title and/or abstract: “aldosterone” AND “bone”, “skeleton”, “osteoporosis”, “fracture”, “calcium”, “parathyroid”, “DXA”, “osteocalcin”, “P1NP”, “alkaline phosphatase”, “bone marker”, “trabecular bone score” or “FRAX”. Exclusion criteria were: in vitro or animal studies, reviews, case reports or series. We screened 1027 articles according to the mentioned strategy, analyzed the papers through the key words, excluded the duplicates, and finally included 23 studies (Figure 2).

## 2. Study-Sample-Based Analysis

These studies (n = 23) were of various designs: 13 of case-control type, 3 cross-sectional, 5 prospective, 1 observational cohort, and 1 retrospective study. The smallest study included 10 patients, while the largest was of 2533 patients. A total of 3965 subjects, aged between 38 and 64, with a mean age 56.75 years (female-to-male ratio of 1.05) were registered.

The assessments provided within these studies were as follows: 9 studies addressed dual-energy X-ray absorptiometry (DXA), another study pointed out a bone microarchitecture evaluation underlying trabecular bone score (TBS), and 7 studies investigated the bone turnover markers (BTM) profile. Moreover, 14 studies followed the subjects after adrenalectomy versus medical treatment, and 21 studies addressed secondary hyperparathyroidism in PA patients (Table 1).

## 3. PA: Mineral Metabolism and Fracture Risk

### 3.1. Bone Status

#### 3.1.1. DXA Assessment in Individuals Confirmed with PA

As mentioned, nine studies examined the association between bone mineral density (BMD) and PA (eight case-control studies and one cross-sectional analysis); these studies provided two types of approaches: BMD results in patients with PA versus controls or osteoporosis/osteopenia prevalence (n = 8) [44,45,46,48,50,52,57,60] or PA prevalence among subjects with osteoporosis (n = 1) [51].

Except for one study [60], BMD was evaluated based on central DXA [44,45,46,48,50,51,52,57,60]. A total of 531 patients underwent DXA; the female-to-male ratio was 0.94 (an average age of 54.48 years). Salcuni et al. [44] found the highest prevalence of osteoporosis among PA patients of 72.7% (N = 11, mean age of 56.0 ± 9.3 years) [44]. The lowest prevalence of osteoporosis was of 10.5% on 73 PA subjects (female-to-male ratio of 0.43; an average age of 52.5 ± 11.2 years) [46].

In three studies, the PA group had lower BMD compared to non-PA patients [44,46,60]; similar BMD between PA and non-PA individuals was found in another three studies [48,50,52]. Additionally, a cohort showed no statistically significant different BMD at the lumbar spine and femoral neck between unilateral and bilateral PA [57]. Ceccoli et al. [45] reported in 40 patients with PA an improvement of lumbar spine BMD after treatment of aldosterone excess (*p* < 0.005) [45].

The earliest study we were able to identify was published in 2012, describing the association between PA and low BMD. Z-score-based BMD (at all central sites) was lower in the PA group when compared to the control group (lumbar spine: *p* = 0.003; femoral neck: *p* = 0.011, and total neck *p* = 0.012). Additionally, the prevalence of osteoporosis was higher in PA versus controls (72.7% versus 20.0%, *p* = 0.015). PA patients had a risk of osteoporosis according to an odds ratio (OR) of 15.4 (95% CI: 1.83–130; *p* = 0.012) [44]. This aspect was confirmed by another study that also evaluated the impact of aldosterone excess on DXA results (73 PA patients versus 73 subjects with essential hypertension versus 40 healthy controls); PA individuals had a statistically significant higher prevalence of osteopenia and osteoporosis (38.5% and 10.5%) than the second group (28% and 4%), respectively, the third group (25% and 5%) [46].

On the contrary, other studies did not confirm a lower BMD in PA versus non-PA subjects, neither a higher prevalence of osteoporosis in these patients. For instance, Notsu et al. [48] showed that lumbar spine and femoral neck BMD were similar in 56 PA patients compared with 56 (non-PA) controls. These results did not exclude a potential aldosterone excess influence on bone microarchitecture as reflected by TBS, and not by BMD [48]. Similarly, Kim et al. [50] suggested that aldosterone may trigger bone loss by deteriorating bone microstructure rather than by reducing the BMD; therefore, BMD was similar between 72 PA patients and 335 subjects diagnosed with non-functional adrenal incidentalomas [50].

Alternatively, quantitative computed tomography (QCT) was used to reflect trabecular bone micro-architectural effects (QCT being an effective tool to measure the volumetric BMD that allows the assessment of volume density of the cancellous and cortical bone). BMD (as measured by QCT) was lower in PA patients versus non-PA controls (141.9 ± 34.0 versus 158.9 ± 55.9 g/cm^3^, *p* = 0.047). Additionally, Lv et al. [60] found a similar prevalence of osteopenia between the two mentioned groups: 31.6% (PA group) versus 30% (the patients with essential hypertension) [60] (Table 2).

On the other hand, Salcuni et al. [51] evaluated the prevalence of PA among patients with osteoporosis or osteoporotic fractures and found ratios of 5.2% and 6.9%, respectively [51] (Table 3).

#### 3.1.2. TBS Evaluation in Patients Diagnosed with PA

TBS, a useful and practical indicator of bone microarchitecture, serves as a fracture risk predictor independently of BMD. TBS seems to be a better pointer than DXA-BMD in PA patients according to Kim et al. [50]. TBS was statistically significantly lower in females with PA (N = 72) than controls (N = 335) after adjustment for confounder factors (*p* = 0.007). The authors show that plasma aldosterone concentration was correlated with a lower TBS in women, but not in men regardless of BMD [50]. Another study reported a higher prevalence of fractures in the PA group versus non-PA, but similar BMD at any site [48]. Overall, the level of statistical evidence concerning TBS assessment in PA remains low (n = 1), and further studies are necessary to pinpoint if PA has a greater impact on bone microarchitecture rather than BMD in adults.

### 3.2. PA and BTMs Profile

BTMs were assessed in seven studies (four case-control, two prospective studies, and one observational longitudinal cohort study), a total of 388 patients (female-to-male ratio of 1.06, and mean age of 52.3 years) [41,43,45,48,52,56,60]. Mostly, the following BTM were analyzed: osteocalcin, procollagen I N-terminal propeptide (PINP) and bone alkaline phosphatase (bALP); respectively, bone resorption markers: urinary deoxypyridinoline (Ur-DPD), urine type I collagen cross-linked N-telopeptide (Ur-NTX), tartrate-resistant acid phosphatase 5b (TrAP), and C-terminal telopeptide of collagen type I (CTX)/CrossLaps. Loh et al. [52] found that both CTX and P1NP levels were statistically significant higher in the PA group compared to controls (*p* = 0.005, and *p* = 0.045) on a small cohort (N = 18, female-to-male ratio of 7:11, with an average age of 50 years) [52]. Also, Adolf et al. [56] identified a slight increase in osteocalcin in 36 menopausal females with PA compared to controls (*p* = 0.023), while the TrAP value was unaltered (*p* = 0.189) [55]. Ceccoli et al. [45] reported that serum CTX was higher and bALP was lower in subjects with PA (N = 116) compared with those diagnosed with essential hypertension (N = 110), but these data did not reach a statistical significance [45]. Similarly, Notsu et al. [48] revealed similar Ur-NTX levels in 56 patients with PA versus 56 controls [48], while two other studies found the same results with regard to Ur-DPD [41,43].

These heterogeneous aspects regarding BTM based on the cross-sectional data were associated with changes in the BMT profile during follow-up from different perspectives. For instance, one study showed a statistically significant reduction in CTX at 3 months following PA therapy (*p* = 0.012); however, bALP remained unchanged [52]. Another study revealed that patients with bilateral adrenal disease undergoing MRA treatment were associated with a statistically significant decrease in osteocalcin (*p* = 0.018), PINP (*p* = 0.007), bone ALP (*p* = 0.004), and TrAP (*p* = 0.028) after 1 year of follow-up, unlike patients who underwent an adrenalectomy [56]. This aspect might suggest that MRA might negatively interfere with bone formation. Yet, Ceccoli et al. [45] studied a cohort of PA patients (40% of them having a unilateral disease) and detected similar BTMs (CTX-I, and bALP) after 24 months of follow-up [45] (Table 4).

### 3.3. Prevalent (Osteoporotic) Fractures among the Patients with PA

Interestingly, despite rather equivocal results with respect to BMD changes in PA, the fracture prevalence was found to be increased in PA patients according to most of the five studies we identified to specifically address this issue (four case control studies and one cross-sectional cohort), with a total of 2725 patients (female-to-male ratio of 1.16; mean age 56.13 years) being assessed [44,48,49,51,57].

The highest prevalence of vertebral fractures was of 45.5% (N = 11 patients with PA) versus 13.3% in non-PA subjects (N = 15; *p* = 0.095) [44]. Also, an analysis revealed a vertebral fracture prevalence of 45% in the PA group when compared to the non-PA group of 23% (*p* = 0.05). Severe vertebral fracture prevalence was statistically significantly higher in PA subjects versus controls (23% versus 4%, *p* = 0.01). PA was found to be a risk factor for vertebral fractures (OR of 3.13; 95%CI: 1.30 to 7.51, *p* = 0.05) independently of blood pressure, glycated hemoglobin, and lipid profile. This aspect seemed to be correlated with microarchitecture damage rather than BMD changes [48].

Similarly, Umakoshi et al. [57] showed that PA subjects (N = 113) had a higher prevalence of vertebral fractures than those without PA (N = 58) of 29% versus 12% (*p* = 0.011). Moreover, subjects with unilateral adrenal disease (N = 37) had a higher prevalence of the same fractures than bilateral PA (N = 76), of 46% versus 20% (*p* = 0.021). In this study, all vertebral fractures in PA patients were asymptomatic. Unilateral PA was an independent risk factor for vertebral fractures (OR = 3.16; 95% CI: 1.12–8.92; *p* = 0.017) regardless of age and sex. Patients with vertebral fractures tended to be older than those without them, but only a tendency of statistical significance was registered (*p* = 0.087) [57].

The incidence rate of fractures (at any site) was of 14.4 per 1000 person-years (PA patients) while individuals with essential hypertension had a rate of 8.3 per 1000 person-years; the prevalence of osteoporotic fractures was 2.9% versus 1.7% (*p* < 0.001), according to a large cohort of 2533 patients with PA [49]. Vertebral fractures were associated with lumbar BMD (OR = 1.71; 95% CI: 1.30–2.25, *p* = 0.001), but not with presence of PA (OR = 3.22; 95% CI: 0.90–12.20, *p* = 0.071) [51] (Table 5).

### 3.4. Mineral Metabolism Assessment in PA: Focus on PTH Levels

According to the mentioned methods, we identified that 21 studies provided PTH values in PA patients (11 case-control studies, 3 cross-sectional studies, 5 prospective studies, 1 observational study, and 1 retrospective study); overall, mineral metabolism was assessed in 1360 patients (female-to-male ratio of 0.883; an average age of 51.67 years). The sample size varied between 10 and 242 individuals per study. Except for 1 study, 20 studies found statistically significant changes in some parameters belonging to mineral metabolism (especially calcium and PTH) in PA versus non-PA subgroups [38,39,40,41,42,43,44,45,46,47,48,51,52,53,54,55,56,57,58,59,60].

Evidence from German Conn’s Registry showed that 54% of the PA patients had secondary hyperparathyroidism [53]; another study reported that 37% of the PA subjects presented an abnormally high PTH level [59]; early studies also suggested a PTH elevation amid PA [38]. Rossi et al. [39] showed that serum intact PTH was increased in PA patients versus subjects with normal blood pressure (*p* < 0.01) while ionized serum calcium was statistically significantly lower (*p* < 0.01) with similar levels of 24 h urinary calcium. An increase in ionized serum calcium and a decrease in PTH was associated with spironolactone administration (*p* < 0.001) and surgical treatment (*p* < 0.05) [39].

Lenzini et al. [54] studied the role of the renin-angiotensin-aldosterone system in PTH regulation. In primary cultures of human parathyroid cells, aldosterone and angiotensin II increased PTH secretion by acting through MRs and angiotensin II type 1 receptors. This further supported the idea that mild hyperparathyroidism is a characteristic of PA and that it may potentially be corrected by adrenalectomy [54]. Recent data suggest that PTH plays a role in PA by triggering and maintaining aldosterone excessive secretion and further stimulating adrenocortical cell proliferation, but more evidence is necessary [41].

Based on our analysis, three studies showed an abnormal mineral metabolism in PA as reflected by higher PTH levels, lower serum calcium, and increased 24 h urinary calcium output when compared to controls. For instance, the control group was represented by subjects with essential high blood pressure (N = 110, mean age of 55 years, with female predominance) in one study; these patients had an increased serum calcium and decreased 24 h urinary calcium versus PA subjects (N = 116, with an average age of 51.6 years, and a slightly higher male predominance) [45]. Another analysis showed statistically significantly lower plasmatic total calcium levels (*p* < 0.001) and higher calcium excretion values (*p* < 0.001) in association with increased plasma PTH levels (*p* < 0.001) in PA subjects versus individuals diagnosed with essential hypertension and healthy controls [46]. Similar data were reported by Jiang et al. [47] (*p* < 0.001, *p* < 0.001, and *p* < 0.001) [47], and by Loh et al. [52] concerning lower serum Ca levels (*p* < 0.013) and increased PTH (*p* < 0.027) compared to individuals with essential hypertension (24 h urinary calcium was not assessed in this particular cohort) [52]. Another two studies showed the same serum calcium and PTH profile, but no statistically significant difference was confirmed regarding urinary calcium output [41,42]. A cross-sectional study (PA: N = 10, and essential hypertension: N = 182) found that PA contributed to secondary hyperparathyroidism, independently of vitamin D level (as reflected by serum 25-hydroxyvitamin D) [42]. Of note, two other studies found higher PTH and 24 h urinary calcium, but no different serum calcium in PA group when compared with different controls [44,60].

Tuersun et al. [55] showed that the prevalence and the severity of abdominal aortic calcifications was elevated in the PA population (N = 156) versus controls (N = 156) represented by subjects with essential hypertension in this instance (*p* = 0.028). Plasma aldosterone concentration and PTH were positively associated with abdominal aortic calcifications in PA patients (OR = 1.235, 95% CI: 1.100–1.373, *p* < 0.001; respectively, OR = 1.038, 95% CI: 1.013–1.064, *p*= 0.002) [55].

A single study (published in 2019) showed among 503 patients (retrospective PA group) a prevalence of 1.2% with regard to primary hyperparathyroidism, while this prevalence was 2.1% in the (prospective PA group). Overall, the prevalence of secondary hyperparathyroidism was 54.6% (in a population aged between 42 and 56 years, an average of 47) [53]. Further evidence is necessary to highlight the association with primary PTH increase in PA.

PTH was found useful to distinguish between patients with unilateral versus bilateral adrenal disease (PTH of 113.4 ± 45.7 ng/L versus 81.7 ± 29.9 pg/mL, *p* = 0.026) in one study; a value of PTH at 80 ng/mL was associated with a sensitivity and a specificity of 74% and 82%, respectively, to identify secondary hyperparathyroidism in PA [43]. Another three studies did not identify a difference regarding PTH levels among these two mentioned subgroups of uni/bilateral adrenal involvement [47,57,59].

A 25-hydroxyvitamin D assay was provided in 13 studies [41,42,43,44,45,46,47,52,53,55,56,58,60]. The values did not reach statistical significance when comparing PA patients with healthy controls [44,60] or with persons diagnosed with essential high blood pressure based on eight studies [42,43,44,45,47,52,55,60]; a case-control study published in 2012 showed that pre-operatory serum vitamin D levels remained unchanged after adrenalectomy (N = 31) [41]. One uncontrolled cohort identified a mean 25-hydroxyvitamin value of 20 ng/mL in PA [53]. However, three studies pointed out differences amid PA, as following: subjects confirmed with PA (N = 73) had statistically significant lower levels when compared to individuals with essential hypertension (N = 73) or healthy controls (N = 40): 17.8 ng/mL versus 32.9 ng/mL, respectively, 23.8 ng/mL (*p* < 0.001 for any) [46]; unexpectedly, 36 menopausal women (N = 36) had higher 25-hydroxyvitamin D than the controls (mean values of 28.9 ng/mL versus 19 ng/mL, *p* = 0.021) [56], while another paper from 2020 showed that baseline vitamin D values were statistically significantly increased during follow-up (21.8 ng/mL versus 29.2 ng/mL, *p* < 0.001) [58] (Table 6).

## 4. Discussion

### 4.1. Dynamics of the Mineral Metabolism and DXA Results in Treated PA Patients

The follow-up data among the mentioned studies represent an inhomogeneous spectrum with respect to three main points to our awareness: firstly, some studied analyzed the effect of therapeutic intervention for PA in terms of (adrenal) surgery or medical treatment such as MRA; secondly, the panel of assessments that were under longitudinal check-up varied from serum and urinary levels of calcium, PTH, BTMs, and BMD—DXA [39,41,42,43,44,45,47,52,53,54,59]. A third point is represented by the specific intervention for osteoporosis and fracture risk reduction in PA subjects, but this aspect remains theoretical since we did not identify any study to particularly address this issue so far.

Overall, 14 studies with various longitudinal data with respect to mineral metabolism and fracture risk evaluation in PA (6 case-control studies, 5 prospective studies, 2 observational studies and 1 retrospective study) and a total of 874 patients being assessed (female-to-male of 0.88, with a mean age of 54 years; between 10 and 242 individuals per study) were investigated. Among these, 11 studies confirmed a statistically significant correction of high PTH after surgical or medical treatment of PA [39,41,42,43,44,45,47,52,53,54,59], and 8 studies also showed that serum calcium levels increased to normal after treatment [38,39,41,43,44,47,53,56]. BMD improvement was not unanimously confirmed after intervention for PA [43,45,52,56].

Post-adrenalectomy changes in mineral metabolism were initially reported in 1985 (based on our PubMed strategy of research): postoperatively, total serum calcium levels increased in six patients diagnosed with unilateral tumors (*p* < 0.01) [38]. Subsequently, Rossi et al. [39] showed an increased serum ionized calcium level and a decreased PTH level after both MRA (spironolactone) administration (*p* < 0.001) and adrenalectomy (*p* < 0.05) [39]. Maniero et al. [41] found that adrenal removal normalized PTH levels (*p* = 0.002) and raised the serum ionized calcium level (*p* < 0.001) in 44 patients with single adrenal disease [41]. After treatment of PA with either adrenal surgery or MRA, PTH concentrations decreased (*p* = 0.023) according to another cohort [42]; PTH was corrected by adrenalectomy (*p* = 0.02) in a group of 46 PA subjects with unilateral disease, in association with a statistically significant decrease in 24 h urinary calcium excretion (*p* = 0.038) [43].

Similarly, 9 out of 11 PA patients included in a small-sized study experienced a statistically significant reduction in urinary calcium output at 6 months after the initiation of specific PA treatment (surgery or spironolactone) (*p* < 0.01), respectively, of PTH (*p* < 0.01); additionally, 5 out of 11 PA patients were associated with a significant lumbar BMD increase at 1 year after starting PA therapy [44]. A BMD elevation was confirmed at the lumbar spine, total hip, and femoral neck in 40 PA subjects after 24 months of post-intervention follow-up in association with a tendency of CTX decrease and bALP increase (N = 16 individuals underwent an adrenalectomy, and N = 24 subjects were treated with MRA) [45]. Also, serum CTX was reduced by 20% (*p* = 0.012) and P1NP decreased by 18% (*p* = 0.036) after unilateral adrenalectomy (N = 3) or MRA treatment (N = 12) based on other data [52]. Therefore, bone turnover may be improved by MRA or adrenal surgery, but the results are limited to relatively small studies [44,45,52].

### 4.2. Unilateral versus Bilateral Adrenal Disease in PA: Mineral and Bone Reflections

As presented, some studies provided data with respect to unilateral versus bilateral PA. This may have a direct or indirect bone reflection depending on different genetic backgrounds and even clinical panels, but the differences lie mostly in management, with unilateral tumors being mostly referred to adrenalectomy, while bilateral disease was followed under MRA exposure. Whether adrenalectomy or medical therapy have distinct skeleton effects is still an open issue.

One study suggested a quite opposite approach, namely PTH serving as a discriminator factor between unilateral and bilateral adrenal involvement in PA; a level above 80 pg/mL may indicate the need to perform adrenal venous sampling for further differential diagnosis between these subtypes [43]. Whether vertebral fracture prevalence is higher in unilateral versus bilateral lesions is relatively difficult to confirm at this point [48], although one study investigated found this result (46% versus 20%, *p* = 0.021) [57].

MRA therapy for 1 year in bilateral PA (N = 18) was associated with BTM decreases such as in osteocalcin (*p* = 0.018), PINP (*p* = 0.007), bALP (*p* = 0.004), and TrAP (*p* = 0.028), but this was not the case following adrenalectomy in unilateral PA (N = 18). We can only speculate that MRA therapy (spironolactone) has an additional bone-protective effect [56].

A few studies showed an increase in PTH levels in unilateral versus bilateral lesions with a reversible pattern following the management of aldosterone over-production [42,45,53,55]. On the other hand, some authors found a similar mineral metabolism profile (for instance, calcium and PTH) among these two PA subtypes [46,47,59].

### 4.3. Why Bone Considerations Next to the Aldosterone Excess?

Most of the mentioned data highlighted secondary hyperparathyroidism in individuals with PA (single-tumor-related PA or association with bilateral adrenal disease). While BMD and even BTMs analysis left us with no clear picture, a higher prevalence of the vertebral fractures in PA groups versus different controls was identified in adult patients. Moreover, TBS seems a promising tool, but limited data have been published so far, while improvement of mineral metabolism (especially serum calcium and PTH) and fracture risk parameters might be registered under surgical or medical therapy for PA.

The underlying mechanisms might include the fact that negative calcium balance through higher urinary/fecal calcium excretion causing hypocalcemia induces secondary hyperparathyroidism, with consecutive bone resorption in PA [61,62]. Also, MRs from human bone cells may be activated by aldosterone excess [35]. Recent studies supported a bidirectional functional link between the adrenocortical zona glomerulosa and the parathyroid gland, MR being present in the parathyroid gland whereas the PTH receptor type 1 was identified in the adrenal gland [63,64]. Primary cultures of human parathyroid cells showed that both aldosterone and angiotensin II may stimulate PTH secretion via angiotensin II type 1 receptors and MR [54,65]. Moreover, oxidative stress might play a role in bone remodeling [66]. Lv et al. [60] showed a higher superoxide dismutase status (*p* = 0.011) in the PA group that could act as an important indicator of oxidative stress in these patients with potential negative impact on BMD [60]. Common pathways of aldosterone signaling and bone strength (involving various genes such as *NR3C2*, *PIK3R1*, *PRKCH*, and *SCNN1B*) might explain bone and mineral metabolism changes in PA [29]. Moreover, cortisol co-secretion in PA has been reported in 4% to 77% of patients with PA; this might cause supplementary bone damage in addition to a higher cardio-metabolic risk through glucocorticoid receptors [67,68,69,70,71].

Another aspect relates to PA management and potential bone health benefits [56,72,73]. Spironolactone may reduce BTMs, as previously shown [56]. Its anti-mineralocorticoid action causes the tubular reabsorption of calcium, thus higher serum calcium levels and a decrease in PTH, in accordance with the effects of thiazide diuretics [74]. Another point is represented by the reduced urinary excretion of magnesium and potassium with potential bone-protective effects in patients receiving MRA [75]. Also, experimental studies found that eplerenone reduced glucocorticoid-induced osteopenia [76]. In addition, vitamin D receptor activity might be correlated with aldosterone excess in PA and interfere with secondary hyperparathyroidism; thus, vitamin D deficiency should be adequately replaced [77,78]. On the other hand, with respect to specific anti-osteoporotic intervention in PA, so far, the traditional management is applicable in these cases since no specific interventional studies addressed the matter of PA-related secondary osteoporosis.

### 4.4. Glucose Profile Anomalies in PA: How about the Fracture Risk?

Perhaps the co-presence of glucose anomalies in PA might explain the heterogeneous panel of BMD results [41,43,45,48,52,56,60] and the potential use of TBS [51] to assess fracture risk, particularly in menopausal women. Glucose metabolism anomalies, regardless the co-diagnosis of cortisol excess, affects up to 25% of PA patients, and it should be taken into consideration not only to address the cardiovascular and metabolic risk, but also the fracture risk, as similarly seen in type 2 diabetes mellitus [79,80,81]. So far, according to our methods of research, we did not identify specific studies to focus on the BMD—TBS profile in the diabetic versus non-diabetic subgroups of PA, and this topic needs a further extension.

Since its release in daily practice, TBS has found its way among different pathogenic forms of diabetes mellitus, not only of type 2, but also in relationship with endocrine tumors such as acromegaly, endogenous Cushing’s syndrome, or primary hyperparathyroidism [82,83,84,85,86]. Of note, primary hyperparathyroidism has been reported in 1.2% to 2.1% of the subjects confirmed with PA according to the German Conn’s Registry [53]. On the other hand, primary hyperparathyroidism may embrace associations with other types of adrenal tumors as seen in multiple endocrine neoplasia type 1 and 2 underlying well known pathogenic variants of *MEN1* and *RET* [87,88,89,90,91]. Whether a subgroup of patients diagnosed with PA also displaying a synchronous parathyroid tumor harbor a common genetic background is yet to be discovered.

### 4.5. From Facts to Further Expansion

There are several clusters of data regarding mineral metabolism and fracture risk in PA. One of them involves central DXA evaluation. Controversies remain as to whether or not BMD alone is sufficient to adequately assess BMD and predict osteoporotic fracture risk in PA. An interesting observation was the fact that plasma renin activity was inversely correlated with TBS only in females, but not in males, but BMD was not statistically significant different between PA cases and controls in both sexes according to one mentioned study [50]. As a lumbar DXA derivate, we mention that the use of TBS should be particularly encouraged in menopausal women with PA-related glucose profile anomalies [92,93].

Another cluster involves vertebral fractures in PA. Grossly, one out of 2 to 5 adults diagnosed with PA display prevalent vertebral fractures (as shown by one study, with mostly an asymptomatic presentation) [44,48,49,51,57]; that is why screening X-rays of the thoracic-lumbar spine profile should be taken into consideration, particularly in cases with other well-known fracture risks such as older age, larger duration of menopausal period, diabetes mellitus, prior fragility fracture, and increased risk of fall due to blood pressure and glycaemia variations, and hypovitaminosis D. Moreover, a collateral hypothesis suggested a higher risk of sarcopenia in females with PA via aldosterone excess or metabolic components, a condition that is prone to fall as part of the general fracture risk panel [94,95,96].

A third group of interest in PA—bone interplay is represented by BTM assays. A heterogeneous BTM picture was described [41,43,45,48,52,56,60], but this might not come as a surprise since the diabetes contribution blunts BTM values; there are already well-known inter-individual and intra-individual BTM variations, while the mentioned BTM studies covered a 2-decade period and the types of BMT tests suffered a great variation over this time [97,98].

A fourth category is represented by not only lower calcium levels, but also mostly by secondary hyperparathyroidism in PA. This subgroup of patients (that might be up to half of the PA subjects) [53] seems at higher cardiovascular risk; the condition affects more cases of unilateral adenomas than bilateral hyperplasia, and it remits upon specific therapy for PA; vitamin D deficiency is an additional contributor, but aldosterone excess exhibits its own effect on parathyroid cells independently of serum 25-hydroxyvitamin D.

Overall, larger longitudinal trials are necessary to point out the role of DXA, TBS, and X-ray scan as screening tools for vertebral fractures, as well as sarcopenia assessment amid PA. Of note, a subgroup of patients diagnosed with PA might be actually at higher risk of osteoporotic fractures, particularly in relationship with traditional panels with respect to menopausal status and older age, and with particular aspects in PA such as the co-presence of cortisol excess or secondary diabetes. Whether PTH functions as a surrogate marker to distinguish between unilateral and bilateral disease is yet to be clarified. However, placing the PTH assay among the PA panel of investigations seems a logical step based on the mentioned studies; identifying the subgroup with secondary hyperparathyroidism helps the cardio-metabolic risk assessment as well.

We are aware of the limitations that come with a narrative review; however, for more than 4 decades, the approach of osteoporotic fracture risk and mineral metabolism status in PA involved different parameters and various perspectives; thus, we intended not to restrict the specific criteria according to a systematic review. Also, choosing a lower cut-off (at least 10 PA patients per study) might bring a potential bias, but the topic of PA-bone is far from generous with regard to the level of statistical evidence. Of note, three of the included studies were published before 2000 [38,39,40], but we intended to adequately highlight the potential PA-bone connections according to the manner in which they were studied over time and to pinpoint the fact that the mentioned cross-domain has been a subject of research for more than 3 decades.

Moreover, despite the fact, as mentioned, that there is still enough space for further studies to highlight the current gaps, recently, this trans-disciplinary topic of the bone-adrenal bridge represents a dynamic point of interest, as also reflected by two meta-analyses from 2020 (n = 15) and 2022 (n = 18) [99,100]. Thus, we can only present our considerations through a sample-based study (n = 23, N = 3965), one of the largest analysis on published data so far. Based on these, we expect not only growing evidence in this particular matter in addition to new molecular insights explaining the complex common pathogenic loops, but also an expansion of the practical recommendations on daily basis to address the skeleton profile in PA. Future lines of research might include controlled studies to address the bone impact of adrenal surgery versus medical treatment; longitudinal data with respect to the post-operatory bone outcome; genetic influence underlying both PA and bone damage; distinct fracture risk subgroups among PA individuals; studies to highlight the specific bone influence due to the co-presence of diabetes mellitus and autonomous cortisol secretion in PA; interventional trials to reveal the best anti-osteoporotic intervention strategy in PA; and potential benefits in terms of fracture risk reduction of the medication targeting aldosterone excess as seen in cardiovascular outcomes.

## 5. Conclusions

Published data during a period of almost 40 years revealed 23 studies with 3965 subjects (aged between 38 and 64, with a mean age 56.75 years, and a female-to-male ratio of 1.05) in which a higher PTH in PA versus controls (healthy persons or subjects with essential hypertension) was expected, secondary hyperparathyroidism being associated in almost half of the adults diagnosed with PA. Additionally, mineral metabolism anomalies in PA may include lower serum calcium and higher urinary calcium output, all these three parameters being reversible under specific therapy for PA, regardless of this therapy being medical or surgical. The PA subgroup with high PTH seems at higher cardiovascular risk, while unilateral rather than bilateral disease is prone to this PTH anomaly. Moreover, BMD might show a higher fracture risk only in certain patients, TBS being a promising alternative (with still unknown perspective of diabetes’ influence on DXA-TBS results in PA). However, overall higher fracture prevalence in PA is described in most studies, especially with respect to vertebral fractures, a risk that seems correctable upon aldosterone excess remission. These data recommend PA as a cause of secondary osteoporosis, a treatable one via PA intervention. There is still an area of debate regarding the way to address the BMT profile in PA, with case selection toward specific bone evaluation in everyday practice, and further on, the understanding of the potential genetic influence at the level of bone and mineral complications in PA patients.

## Figures and Tables

**Table 1:** Studies included in the final analysis (n = 23) according to the mentioned strategy (N ≥ 10 patients per study); the display is between 1985 and 2022; the table provides the study design and studied population [38,39,40,41,42,43,44,45,46,47,48,49,50,51,52,53,54,55,56,57,58,59,60].

First Author, Publication Year, Reference Number Study Design	Studied Population	Female-to-Male Ratio (F/M); Age (Years)
Resnick, 1985 [38] observational study	N = 10 PA	F/M = 5/5; mean age: 53 ± 4 y
Rossi, 1995 [39] Case-control study	N1 = 10 PA N2 = 10 normotensive control subjects	N1: F/M = 5/5; mean age: 52.4 ± 12.9 y N2: F/M = 5/5; mean age: 48 ± 12.7 y
Rossi, 1998 [40] Case-control study	N1 = 16 PA N2 = 16 controls	N1: F/M = 8/8; mean age: 50.8 ± 2.7 y N2: F/M = 8/8; mean age: 48.5 ± 2.3 y
Maniero, 2012 [41] Case-control study	N1 = 44 PA N2 = 61 EH controls	N1: F/M = 14/30; mean age: 50 ± 13 y N2: F/M = 30/31; mean age: 50 ± 15 y
Pilz, 2012 [42] Cross-sectional and interventional study	N1 = 10 PA N2 = 182 EH	N1: F/M = 6/4; mean age: 50.1 ± 11.0 y N2: F/M = 107/75; mean age: 50.2 ± 15.7 y
Rossi, 2012 [43] Prospective study	N1 = 46 APA N2 = 12 BAH N3 = 74 EH	N1: mean age: 51 ± 13 y N2: mean age: 45 ± 10 y N3: mean age: 50 ± 14 y
Salcuni, 2012 [44] Case-control study	N1 = 11 PA N2 = 15 controls	N1: F/M = 7/4; mean age: 56.0 ± 9.3 y N2: F/M = 10/5; mean age: 56.7 ± 9.5 y
Ceccoli, 2013 [45] Case-control study	N1 = 116 PA (46 with APA and 70 with BAH) N2 = 110 EH	N1: F/M = 51/65; mean age: 51.6 ± 11 y N2: F/M = 74/36; mean age: 55 ± 10 y
Petramala, 2014 [46] Case-control study	N1 = 73 PA N2 = 73 EH N3 = 40 HS	N1: F/M = 22/51; mean age: 52.5 ± 11.2 y N2: F/M = 38/35; mean age: 55.6 ± 12.4 y N3: F/M = 24/16; mean age: 55.7 ± 6.1 y
Jiang, 2016 [47] Case-control study	N1 = 242 PA N2 = 120 EH	N1: F/M = 112/130; mean age: 49 (41–57 y) N2: F/M = 67/53; mean age: 50 (42–58 y)
Notsu, 2017 [48] Case-control study	N1 = 56 PA N2 = 56 controls	N1: F/M = 31/25; mean age: 58.7 ± 11.1 y N2: F/M = 31/25; mean age: 59.4 ± 11.5 y
Wu, 2017 [49] Case-control study	N1 = 2533 PA N2 = 10,132 EH	N1: F/M = 1357/1176; mean age: 50.55 ± 14.57 y N2: F/M = 5424/4708; mean age: 50.69 ± 17.90 y
Kim, 2017 [50] Case-control study	N1 = 72 PA N2 = 335 controls	N1: F/M = 38/34; mean age (F): 56.4 ± 9.4 y; mean age (M): 57.1 ± 7.5 y N2: F/M = 136/199; mean age (F): 54.4 ± 10.2 y; mean age (M): 54.8 ± 9.8 y
Salcuni, 2017 [51] Case-control study	N1 = 12 PA N2 = 310 controls	N1: mean age: 60.4 ± 13.5 y N2: mean age: 61.1 ± 9.3 y
Loh, 2018 [52] Case-control study Prospective study	N1 = 18 PA N2 = 17 EH	N1: F/M = 7/11; mean age: 50 y (38.0–58.75 y) N2: F/M = 6/11; mean age: 50 y (38.5–61.5 y)
Asbach, 2019 [53] Retrospective-prospective study	Cohort 1 = 503 patients with PA Cohort 2 = 141 prospective PA patients (N = 125 patients with PA from cohort 2 were analysed)	N: F/M = 46/79; mean age: 47 (42–56 y)
Lenzini, 2019 [54] Prospective study	N1 = 27 APA N2 = 15 BAH N3 = 63 EH	N1: F/M = 12/15; mean age: 52 ± 11 y N2: F/M = 6/9; mean age: 52 ± 12 y N3: F/M = 26/37; mean age: 52 ± 10 y
Tuersun, 2020 [55] Cross-sectional study	N1 = 156 PA N2 = 156 EH	N1: F/M = 68/88; mean age: 49.86 ± 8.72 y N2: F/M = 68/88; mean age: 48.99 ± 8.60 y
Adolf, 2020 [56] Observational longitudinal cohort study	N1 = 36 postmenopausal females N2 = 18 controls	N1: F/M = 36/0; mean age: 59 y (53–64 y) N2: F/M = 18/0; mean age: 54 y (44–60 y)
Umakoshi, 2020 [57] Retrospective cross-sectional study	N1 = 37 unilateral PA N2 = 76 bilateral PA N3 = 58 without PA	N1: F/M = 12/25; mean age: 56 ± 14 y N2: F/M = 53/23; mean age: 54 ± 11 y
Ismail, 2020 [58] Prospective interventional study	N = 17 PA	F/M = 9/8; mean age: 42 ± 8 y
Kometani,2021 [59] Retrospective study	N1 = 135 PA	N1: F/M = 66/69; mean age: 52 ± 11 y
Lv, 2022 (60) [49] Retrospective case-control study	N1 = 60 with PA N2 = 60 controls	N1: F/M = 31/29; mean age: 48.8 ± 13.1 y N2: F/M = 31/29; mean age: 48.4 ± 13.8 y

Abbreviations: APA: aldosterone-producing adenoma, BAH: bilateral adrenal hyperplasia, EH: essential hypertension, F: female, HS: healthy subjects, M: male, N: number of patients, PA: primary aldosteronism, y: year (of note, the terms were used according to each original study).

**Table 2:** Studies providing DXA results in patients diagnosed with PA (n = 8 studies); the display is based on publication year starting with the earliest publication in 2012 [44,45,46,48,50,52,57,60].

First Author, Publication Year, Reference Number Study Design Number of Patients	DXA Results (N1) (#)	DXA Results (N2) (#)	Results
Salcuni, 2012 [44] Case-control study N1 = 11 PA N2 = 15 controls	LS-BMD = 0.85 ± 0.18 g/cm^2^ LS (Z-score) = −1.18 ± 0.99 SD FN-BMD = 0.68 ± 0.10 g/cm^2^ FN (Z-score) = −0.85 ± 0.73 SD Osteoporosis prevalence = 72.7%	LS-BMD = 0.98 ± 0.15 g/cm^2^ LS (Z-score) = 0.22 ± 1.12 SD FN-BMD = 0.76 ± 0.08 g/cm^2^ FN (Z-score) = 0.01 ± 0.82 SD Osteoporosis prevalence = 20%	LS/FN BMD/Z-score: N1 < N2 (*p* = 0.003, *p* = 0.011, *p* = 0.012). PA was associated with osteoporosis (OR = 15.4; 95%CI, 1.83–130; *p* = 0.012)
Ceccoli, 2013 [45] Case-control study N1 = 116 PA (46 with APA and 70 with BAH) N1A/B = 40 PA before/after treatment N2 = 110 EH	N1A LS-BMD = 0.95 ± 0.16 g/cm^2^ LS Z-score = −1.3 ± 0.8 SD FN-BMD = 0.84 ± 0.15 g/cm^2^ FN Z-score = −1.1 ± 0.8 SD TH-BMD = 0.95 ± 0.21 g/cm^2^ TH Z-score = −0.5 ± 1.1 SD	N1B LS-BMD = 1.10 ± 0.17 g/cm^2^ LS Z-score = −0.9 ± 0.8 SD FN-BMD = 0.88 ± 0.16 g/cm^2^ FN Z-score = −0.6 ± 0.8 SD TH-BMD = 0.97 ± 0.20 g/cm^2^ TH Z-score = −0.2 ± 1 SD	N1A versus N1B: ↗LS-BMD: *p* < 0.005 ↗LS Z-score: *p* < 0.0001 FN-BMD: *p* = NS ↘FN Z-score: *p* < 0.0001 TH-BMD: *p* = NS ↘TH Z-score: *p* < 0.0001
Petramala, 2014 [46] Case-control study N1 = 73 PA N2 = 73 EH N3 = 40 HS	LS T-score = −0.28 ±1.3 SD LS BMD = 1.01 ± 0.17 g/cm^2^ FN T-score = −0.67 ± 1.1 SD FN BMD = 0.84 ± 0.16 g/cm^2^ Osteoporosis/osteopenia prevalence = 10.5%/38.5% PA-FN BMD/T-score correlation (r = −0.27; *p* < 0.05/r = −0.28; *p* < 0.04)	LS T-score = 0.03 ± 0.6 SD LS BMD = 1.11 ± 0.17 g/cm^2^ FN T-score = −0.29 ± 0.7 SD FN BMD = 0.84 ± 0.12 g/cm^2^ Osteoporosis/osteopenia prevalence = 4%/28%	N3: LS T-score = 0.027 ± 0.8 SD LS BMD = 1 ± 0.09 g/cm^2^ FN T-score = −0.30 ± 0.6 SD FN BMD = 0.81 ± 0.08 g/cm^2^ Osteoporosis/osteopenia prevalence = 5%/25%
Notsu, 2017 [48] Case-control study N1 = 56 PA N2 = 56 controls	LS-BMD = 0.926 ± 0.20 g/cm^2^ LS T-score = −0.52 ± 1.48 SD LS Z-score = 0.22 ± 1.32 SD FN-BMD = 0.681 ± 0.165 g/cm^2^ FN T-score = −1.19 ± 1.02 SD FN Z-score = −0.10 ± 1.14 SD	LS-BMD = 0.971 ± 0.183 g/cm^2^ LS T-score = −1.00 ± 1.75 SD LS Z-score = 0.50 ± 1.19 SD FN-BMD = 0.684 ± 0.130 g/cm^2^ FN T-score = −1.24 ± 1.25 SD FN Z-score = 0.03 ± 1.07 SD	N1 versus N2: LS-BMD: *p* = 0.251 T-score: *p* = 0.251 Z-score: *p* = 0.267 FN-BMD: *p* = 0.907 FN T-score: *p* = 0.798 FN Z-score: *p* = 0.596
Kim, 2017 [50] Case control study N1 = 72 PA N2 = 335 controls	LS BMD similar between N1 and N2 *		
Loh, 2018 [52] Case-control study Prospective study N1 = 18 PA N2 = 17 EH	LS-BMD = 1.042 g/cm^2^ (0.974–1.154) LS Z-score = 0.45 SD (0.0–1.05) FN-BMD = 0.781 g/cm^2^ (0.67–0.963) FN Z-score = 0.05 SD (−0.4–1.78) TN-BMD = 0.965 g/cm^2^ (0.853–1.160) TN Z-score = 0.90 SD (0.18–1.83) DR-BMD = 0.690 g/cm^2^ (0.662–0.779) DR Z-score = −0.35 SD (−1.0–0.25)	LS-BMD = 1.015 g/cm^2^ (0.923–1.154) LS Z-score = 0.50 SD (−0.3–1.45) FN-BMD = 0.841 g/cm^2^ (0.720–0.927) FN Z-score = 0.5 SD (−0.2–1.65) TN-BMD = 1.031 g/cm^2^ (0.875–1.082) TN Z-score = 1.10 SD (0.20–1.60) DR-BMD = 0.727 g/cm^2^ (0.669–0.794) DR Z-score = −0.6 SD (−1.0–1.05)	N1 versus N2: LS-BMD: *p* = 0.807 LS Z-score: *p* = 0.961 FN-BMD: *p* = 0.636 FN Z-score: *p* = 0.59 TN-BMD: *p* = 0.909 TN Z-score: *p* = 0.9 DR-BMD: *p* = 0.463 DR Z-score: *p* = 0.732
Umakoshi, 2020 [57] Retrospective cross-sectional study N1 = 37 U PA N2 = 76 bilateral PA N3 = 58 without PA	LS-BMD = 0.973 ± 0.217 g/cm^2^ FN-BMD = 0.702 ± 0.128 g/cm^2^ N1 + N2 (PA patients): Osteoporosis/osteopenia prevalence = 21%/67%	LS-BMD = 0.897 ± 0.171 g/cm^2^ FN-BMD = 0.676 ± 0.115 g/cm^2^	N1 versus N2: LS-BMD: *p* = 0.096 FN-BMD: *p* = 0.364
Lv, 2022 [60] Retrospective study N1 = 60 PA N2 = 60 controls	BMD = 141.9 ± 34.0 g/cm^3^ ** Osteopenia prevalence = 31.6%	BMD = 158.9 ± 55.9 g/cm^3^ Osteopenia prevalence = 30%	BMD: N1 < N2 (*p* = 0.047) Osteopenia prevalence (*p* = 0.843)

Abbreviations: BMD: bone mineral density; CI: confidence interval; DR: distal radius; EH: essential hypertension; FN: femoral neck; HS: healthy subjects; LS: lumbar spine; N: number of patients; NS: not statistically significant; OR: odds ratio; PA: primary aldosteronism; TH: total hip; U: unilateral; ↗ increased; ↘ decreased; * data not provided; ** BMD was measured by QCT (quantitative computed tomography); (#) values represent mean ± standard deviation.

**Table 3:** Study of PA prevalence among patients with osteoporosis [51].

First Author, Publication Year, Reference Number Study Design, Studied Population	DXA Assessment (*)	PA Analysis among Patients with Osteoporosis
Salcuni, 2017 [51] Case-control study N1 = 12 PA N2 = 310 controls	N1: LS BMD (Z-score) = −0.70 ± 1.25 SD FN BMD (Z-score) = −0.68 ± 0.78 SD TN BMD (Z-score) = −0.26 ± 1.03 SD N2: LS BMD (Z-score) = −0.95 ± 1.25 SD FN BMD (Z-score) = −0.61 ± 0.89 SD TN BMD (Z-score) = −0.51 ± 1.00 SD	Prevalence of PA among patients with osteoporosis/fractures = 5.2%/6.9%

Abbreviations: BMD: bone mineral density; FN: femoral neck; LS: lumbar spine; PA: primary aldosteronism; SD: standard deviation; TN: total neck; * values represent mean ± standard deviation.

**Table 4:** Studies that provided BTMs and calcium metabolism evaluation in subjects confirmed with PA according to our methods; the display starts with the earliest publication date [41,43,45,48,52,56,60].

First Author, Publication Year, Reference Number Study Design, Studied Population	Bone Turnover Markers Profile (*)
Maniero, 2012 [41] Case-control study N1 = 44 PA; N2 = 61 EH controls	N1 versus N2: similar Ur-DPD (*p* = NS)
Rossi, 2012 [43] Prospective study N1 = 46 APA; N2 = 12 BAH; N3 = 74 EH	N1: Ur-DPD/creatinine = 6.18 ± 1.32 nmol/mmoL N2: Ur-DPD/creatinine = 7.03 ± 2.45 nmol/mmoL N3: Ur-DPD/creatinine = 6.11 ± 3.67 nmol/mmoL N1 versus N2 versus N3: similar Ur-DPD (*p* = NS)
Ceccoli, 2013 [45] Case-control study N1 = 116 PA (46 with APA and 70 with BAH); N2 = 110 EH	N1: CTX-I = 603 ± 187 pg/mL; bALP = 10.8 ± 2.9 μg/L N2: CTX-I = 540 ± 321 pg/mL; bALP = 14.8 ± 8.4 μg/L N1 versus N2: similar CTX, bALP (*p* = NS)
Notsu, 2017 [48] Case-control study N1 = 56 PA; N2 = 56 controls	N1: Ur-NTX = 50.8 ± 42.4 nMBCE/mM N2: Ur-NTX = 50.5 ± 95.1 nMBCE/mM N1 versus N2: Ur-NTX (*p* = 0.982)
Loh, 2018 [52] Case-control study Prospective study N1 = 18 PA; N2 = 17 EH	N1: P1NP = 42.59 ng/mL ALP = 70.5 IU/L CTX = 0.406 ng/mL N2: P1NP = 33.95 ng/mL ALP = 64.0 IU/L CTX = 0.277 ng/mL N1 versus N2: P1NP (*p* = 0.045); ALP (*p* = 0.11); CTX (*p* = 0.005)
Adolf, 2020 [56] Observational longitudinal cohort study N1 = 36 postmenopausal females; N2 = 18 controls	N1: osteocalcin = 20.6 ng/mL PINP = 55.1 ng/mL bALP = 17.0 µg/L TrAP = 2.3 U/L N2: osteocalcin = 12.4 ng/mL PINP = 50.1 ng/mL bALP = 17.8 µg/L TrAP = 2.1 U/L N1 versus N2: osteocalcin (*p* = 0.023); PINP (*p* = 0.419); bALP (*p* = 1.000); TrAP (*p* = 0.189)
Lv, 2022 [60] Retrospective case-control study N1 = 60 with PA; N2 = 60 controls	N1: osteocalcin = 13.4 ng/mL PINP = 46.3 ng/mL Crosslaps = 0.5 ng/mL N2: osteocalcin = 13.8 ng/mL PINP = 51.6 ng/mL Crosslaps = 0.4 ng/mL N1 versus N2: osteocalcin (*p* = 0.587); PINP (*p* = 0.979); Crosslaps (*p* = 0.109)

Abbreviations: APA: aldosterone producing adenoma; ALP: alkaline phosphatase; BAH: bilateral adrenal hyperplasia; bALP: bone alkaline phosphatase; CTX-I: C-terminal telopeptide of collagen type I; EH: essential hypertension; N: number of patients; NS: not significant; PA: primary aldosteronism; TrAP: Tartrate-resistant acid phosphatase 5b; Ur-NTX: urine type I collagen cross-linked N-telopeptide; Ur-DPD: Urinary deoxypyridinoline; * values represent mean ± standard deviation.

**Table 5:** Studies that analyzed the fracture prevalence in PA patients; the display is between 2012 and 2020 [44,48,49,51,57].

First Author, Publication Year, Reference Number, Study Design, Studied Groups	Osteoporotic (Vertebral) Fractures Prevalence
Salcuni, 2012 [44] Case control study; N1 = 11 PA; N2 = 15 controls	N1: 45.5% versus N2: 13.3%; *p* = 0.095 (VF)
Wu, 2017 [49] Case-control study; N1 = 2533 PA; N2 = 10,132 EH	N1: 2.9% versus N2: 1.7%; *p* < 0.001 (OF)
Notsu, 2017 [48] Case-control study; N1 = 56 PA; N2 = 56 controls	N1: 45% versus N2: 23%; *p* = 0.05 (VF)
Salcuni, 2017 [51] Case-control study; N1 = 12 PA; N2 = 310 controls	N1: 41.7% versus N2: 21.6%; *p* = 0.07 (VF)
Umakoshi, 2020 [57] Retrospective cross-sectional study; N1 = 37 unilateral PA; N2 = 76 bilateral PA; N3 = 58 without PA	N1: 46%; N2: 20%; N1 + N2: 29%; N3: 12% N1 + N2 > N3; *p* = 0.011 (VF) N1 > N3: *p* = 0.002 (VF)

Abbreviations: EH: essential hypertension; N: number of patients; OF: osteoporotic fracture; PA: primary aldosteronism; VF: vertebral fracture.

**Table 6:** Studies with mineral metabolism assessment (particularly PTH assays) in patients with PA; the display is based on publication date, starting with 1985 [38,39,40,41,42,43,44,45,46,47,48,51,52,53,54,55,56,57,58,59,60].

First Author, Publication Year, Reference Number, Study Design, Studied Population	Calcium Metabolism Assays	PTH Levels
Resnick,1985 [38] Observational study N = 10	Ca = 9.03 ± 0.2 mg/dL (8.80–10.0)	PTH = 645 ± 109 pgeq/L (Normal: 150–375) High PTH: 8 out of 10 patients
Rossi, 1995 [39] Case control study N1 = 10 PA N2 = 10 NC	N1: Ca = 8.94 ± 0.31 mg/dL Ur-Ca = 206 ± 91 mg/24 h N2: Ca = 8.86 ± 0.30 mg/dL Ur-Ca = 182 ± 80 mg/24 h	N1 PTH = 70 ± 24 pg/mL N2 PTH = 41 ± 15 pg/mL (*p* < 0.01)
Rossi, 1998 [40] Case control study N1 = 16 PA N2 = 16 controls	N1: Baseline: Ca = 1.23 ± 0.01 mmol/L Ur-Ca = 8.43 ± 0.68 mg/24 h After saline load: Ca = 1.14 ± 0.02 mmol/L Ur-Ca = 16.79 ± 1.53 mg/24 h N2: Baseline: Ca = 1.24 ± 0.01 mmol/L Ur-Ca = 5.89 ± 0.53 mg/24 h After saline load: Ca = 1.19 ± 0.01 mmol/L Ur-Ca = 8.99 ± 0.88 mg/24 h	N1: Baseline PTH = 47.5 ± 5.1 pg/mL After saline load PTH = 67.1 ± 6.1 pg/mL N2:Baseline PTH = 33.4 ± 3.5 pg/mL After saline load PTH = 40.4 ± 3.9 pg/mL
Maniero, 2012 [41] Case-control study N1 = 44 PA N2 = 61 EH controls	N1: Surgically treated (N = 31) Baseline: Ca = 1.17 ± 0.04 mmol/L Ur-Ca = 7 ± 1.2 mmol/24 h 25OHD = 34 ± 21 nmol/L Follow-up: Ca = 1.22 ± 0.03 mmol/L (*p* < 0.001) Ur-Ca = 5.1 ± 3.2 mmol/24 h (*p* = NS) 25OHD = 38 ± 21 nmol/L (*p* = NS)	N1: Surgically treated (N = 31) Baseline PTH = 118 ± 13 ng/L Follow-up PTH = 76 ± 11 ng/L (*p* = 0.002)
Pilz, 2012 [42] Cross-Sectional study N1 = 10 PA N2 = 182 EH	N1: Ca = 2.26 ± 0.10 mmol/L Ur-Ca to creatinine ratio = 0.33 25OHD = 33.0 ± 23.7 ng/mL N2: Ca = 2.35 ± 0.10 mmol/L (*p* = 0.013) Ur-Ca to creatinine ratio = 0.19 (*p* = 0.094) 25OHD = 30.5 ± 15.0 ng/mL (*p* = 0.748)	N1 PTH = 67.8 ± 26.9 pg/mL N2 PTH = 46.5 ± 20.9 pg/mL (*p* = 0.002)
Rossi, 2012 [43] Prospective study N1 = 46 APA N2 = 12 BAH N3 = 74 EH	N1: Ca = 2.30 ± 0.10 mmol/L Ur-Ca = 5.66 ± 3.4 mmol/24 h 25OHD = 44.6 ± 27.3 nmol/L N2: Ca = 2.38 ± 0.11 mmol/L (N1 versus N2: *p* = 0.051) Ur-Ca = 4.19 ± 2.10 mmol/24 h (N1 versus N2: *p* = NS) 25OHD = 54.4 ± 20.4 nmol/L (N1 versus N2: *p* = NS) N3: Ca = 2.34 ± 0.09 mmol/L (N1 versus N3: *p* = NS; N2 versus N3: *p* = NS) Ur-Ca = 8.53 ± 28.84 mmol/24 h (N1 versus N3: *p* = NS; N2 versus N3: *p* = NS) 25OHD = 47.2 ± 22.0 nmol/L [N1 (or N2) versus N3: *p* = NS)]	N1 PTH = 113.4 ± 45.7 pg/mL N2 PTH = 81.7 ± 29.9 pg/mL (N1 versus N2: *p* = 0.026) N3 PTH = 79.0 ± 30.8 pg/mL (N1 versus N3: *p* < 0.001; N2 versus N3: *p* = NS)
Salcuni, 2012 [44] Case-control study N1 = 11 PA N2 = 15 controls	N1:Ca = 2.20 ± 0.12 mmol/L Ur-Ca = 6.28 ± 1.85 mmol/24 h 25OHD = 32.3 ± 13.0 nmol/L N2: Ca = 2.22 ± 0.11 mmol/L (N1 versus N2: *p* = NS) Ur-Ca = 4.28 ± 1.18 mmol/24 h (N1 versus N2: *p* = 0.002) 25OHD = 35.0 ± 17.8 nmol/L (N1 versus N2: *p* = NS)	N1 PTH = 9.8 pmol/L N2 PTH = 5.3 pmol/L (*p* = 0.001)
Ceccoli, 2013 [45] Case-control study N1 = 116 PA (46 with APA and 70 with BAH) N2 = 110 EH	N1: Ca = 8.9 ± 0.3 mEq/L Ur-Ca = 201 ± 86 mg/24 h 25OHD = 24 ± 15 ng/mL N2: Ca = 9.2 ± 0.6 mEq/L (*p* < 0.05) Ur-Ca = 122 ± 84 mg/24 h (*p* < 0.0005) 25OHD = 26 ± 18 ng/mL (*p* = NS)	N1 PTH = 82.2 ± 33 pg/mL N2 PTH = 56.4 ± 16.4 pg/mL (*p* < 0.05)
Petramala, 2014 [46] Case-control study N1 = 73 PA N2 = 73 EH N3 = 40 HS	N1: Ca = 9.2 ± 0.4 mg/dL Ur-Ca = 242.8 ± 116.7 mg/24 h 25OHD = 17.8 ± 12.5 ng/mL N2: Ca = 9.7± 0.3 mg/dL Ur-Ca = 164.1 ± 84 mg/24 h 25OHD = 32.9 ± 16 ng/mL N3: Ca = 9.4 ± 0.3 mg/dL (N1 versus N2 + N3: *p* < 0.001) Ur-Ca = 154.6 ± 17.3 mg/24 h (N1 versus N2 + N3: *p* < 0.001) 25-OHD = 23.8 ± 12.8 ng/mL (N1 versus N2 + N3: *p* < 0.001)	N1 PTH = 48.9 ± 19.9/mL N2 PTH = 30.7 ± 11.9 pg/mL N3 PTH = 29.1 ± 2.4 pg/mL (N1 versus N2 + N3: *p* < 0.001)
Jiang, 2016 [47] Case-control study N1 = 242 PA N2 = 120 EH	N1: Ca = 2.15 mmol/L Ur-Ca = 5.0 mmol/24 h 25OHD = 28.5 nmol/L N2: Ca = 2.2 mmol/L (*p* < 0.001) Ur-Ca = 4.2 mmol/24 h (*p* < 0.001) 25OHD = 34.0 nmol/L (*p* = 0.06)	N1 PTH = 9.0 pmol/L N2 PTH = 5.7 pmol/L (*p* < 0.001)
Notsu, 2017 [48] Case-control study N1 = 56 PA N2 = 56 controls	N1: Ca = 9.1 ± 0.4 mg/dL N2: Ca = 9.2 ± 0.4 mg/dL (*p* = 0.193)	N1: PTH = 56 ± 37 pg/mL N2: PTH = 47 ± 26 pg/mL (*p* = 0.171)
Salcuni, 2017 [51] Case-control study N1 = 12 PA N2 = 310 controls	N1: Ur-Ca = 7.6 ± 3.2 mmol/24 h N2: Ur-Ca = 4.8 ± 2.5 mmol/24 h (*p* < 0.001)	N1: PTH = 7.3 pmol/L N2: PTH = 5.4 pmol/L (*p* < 0.01)
Loh, 2018 [52] Case-control, prospective study N1 = 18 PA N2 = 17 EH	N1: Ca = 2.26 mmol/L 25OHD = 21.39 nmol/L N2: Ca = 2.32 mmol/L (*p* = 0.013) 25OHD = 23.33 nmol/L (*p* = 0.424)	N1: PTH = 4.26 pmol/L N2: PTH = 2.72 pmol/L (*p* = 0.027)
Asbach, 2019 [53] Retrospective-prospective study N = 125 PA	Ca = 2.4 mmol/L 25OHD = 20.9 ng/mL	iPTH = 67.7 pg/mL
Lenzini, 2019 [54] Prospective study N1 = 27 APA N2 = 15 BAH N3 = 63 EH	NA	N1: PTH = 34.6 ± 14.2 ng/L N2: PTH = 31.6 ± 12.1 ng/L N3: PTH= 25.9 ± 8.3 ng/L (N1 versus N3: *p* < 0.0001)
Tuersun, 2020 [55] Cross-sectional study N1 = 156 PA N2 = 156 EH	N1: Ca = 2.31 ± 0.13 mmol/L Ur-Ca = 5.77 ± 2.64 mmol/24 h 25OHD = 19.40 ± 8.55 ng/mL N2: Ca = 2.26 ± 0.30 mmol/L (*p* = 0.323) Ur-Ca = 5.03 ± 2.30 mmol/24 h (*p* = 0.011) 25OHD = 18.57 ± 8.02 ng/mL (*p* = NS)	N1: PTH = 58.28 ± 27.52 pg/mL N2: PTH = 41.73 ± 20.21 pg/mL (*p* < 0.001)
Adolf, 2020 [56] Observational longitudinal cohort study N1 = 36 menopausal females N2 = 18 controls	N1: Ca = 2.4 mmol/L Ur-Ca = 4.6 mmol/24 h 25OHD = 28.9 ng/mL N2: Ca = 2.5 mmol/L (*p* = 0.255) 25OHD = 19.0 ng/mL (*p* = 0.021)	N1: PTH = 53.5 mg/dL N2: PTH = 66.3 mg/dL (*p* = 0.370)
Umakoshi, 2020 [57] Retrospective cross-sectional study N1 = 37 unilateral PA N2 = 76 bilateral PA	N1: Ca = 9.2 ± 0.4 mg/dL N2: Ca = 9.2 ± 0.3 mg/dL (*p* = 0.287)	N1: PTH = 64 ± 24 pg/mL N2 PTH = 58 ± 29 pg/mL(*p* = 0.340)
Ismail, 2020 [58] Prospective interventional study N = 17 PA	Baseline: Ca = 2.34 ± 0.1 mmol/L Ur-Ca = 3.7 ± 2.2 mmol/24 h 25OHD = 21.8 ± 12.4 ng/mL Follow-up: Ca = 2.37 ± 0.1 mmol/L (*p* = 0.2) Ur-Ca = 3.2 ± 1.4 mmol/24 h (*p* = 0.36) 25OHD = 29.2 ± 13.7 ng/mL (*p* < 0.001)	Baseline iPTH = 4.74 ± 1.4 pmol/L Follow-up iPTH = 4.43 ± 1.6 pmol/L (*p* = 0.45)
Kometani, 2021 [59] Retrospective study N1 = 135 PA	Ca = 9.2 ± 0.4 mg/dL	PTH = 60 ± 29 pg/mL 37% of PA patients had a higher PTH
Lv, 2022 [60] Retrospective case-control study N1 = 60 with PA N2 = 60 controls	N1: Ca = 2.30 ± 0.10 mmol/L Ur-Ca 24 h = 6.0 mmol/24 h 25OHD = 43.0 nmol/L N2: Ca = 2.33 ± 0.19 mmol/L (*p* = 0.363) Ur-Ca24 h = 4.8 mmol/24 h (*p* < 0.001) 25OHD = 48.6 nmol/L(*p* = 0.083)	N1: iPTH = 49.5 pg/mL N2: iPTH = 36.0 pg/mL (*p* < 0.001)

Abbreviations: APA: aldosterone producing adenoma; BAH: bilateral adrenal hyperplasia; Ca: total serum calcium; EH: essential hypertension; HS: healthy subjects; iPTH: intact parathyroid hormone; PTH: parathyroid hormone; N: number of patients; NS: non-significant; NC: normotensive control patients; NA: not available; PA: primary aldosteronism; Ur-Ca: 24 h urinary calcium; 25OHD: 25-hydroxyvitamin D; red colour highlights the specific description of a certain subgroup.

## Data Availability

Not applicable.

## Abbreviations

BTMs	bone turnover markers
BMD	bone mineral density
bALP	bone alkaline phosphatase
CI	confidence interval
CTX-I/CrossLaps	C-terminal telopeptide of collagen type I
DXA	dual-energy X-ray absorptiometry
24 h	24 hours
iPTH	intact parathyroid hormone
MRA	mineralocorticoid receptor antagonists
OR	odds ratio
PA	primary aldosteronism
PTH	parathyroid hormone
P1NP	procollagen I N-terminal propeptide
OF	osteoporotic fracture
QCT	quantitative computed tomography
TBS	trabecular bone score
TrAP	Tartrate-resistant acid phosphatase 5b
WHO	World Health Organization
VF	vertebral fracture
Ur-NTX	urine type I collagen cross-linked N-telopeptide
Ur-DPD	urinary deoxypyridinoline
